# Ecotropic viral integration site 1 regulates *EGFR* transcription in glioblastoma cells

**DOI:** 10.1007/s11060-019-03310-z

**Published:** 2019-10-15

**Authors:** Asako Mizuguchi, Shinji Yamashita, Kiyotaka Yokogami, Kazuhiro Morishita, Hideo Takeshima

**Affiliations:** 1 Department of Neurosurgery, Faculty of Medicine, University of Miyazaki, 5200, Kiyotake-cho, Kihara, Miyazaki-shi, Miyazaki, 889-1601 Japan; 2 Department of Tumor and Cellular Biochemistry, Faculty of Medicine, University of Miyazaki, 5200, Kiyotake-cho, Kihara, Miyazaki-shi, Miyazaki, 889-1601 Japan

**Keywords:** Ecotropic viral integration site-1, Epidermal growth factor receptor, Glioblastoma, Transcription factor, Tyrosine kinase receptor

## Abstract

**Purpose:**

Ecotropic viral integration site-1 (EVI1) is a transcription factor that contributes to the unfavorable prognosis of leukemia, some epithelial cancers, and glial tumors. However, the biological function of EVI1 in glioblastoma multiforme (GBM) remains unclear. Based on microarray experiments, EVI1 has been reported to regulate epidermal growth factor receptor (*EGFR*) transcription. Signal transduction via EGFR plays an essential role in glioblastoma. Therefore, we performed this study to clarify the importance of *EVI1* in GBM by focusing on the regulatory mechanism between EVI1 and *EGFR* transcription.

**Methods:**

We performed immunohistochemical staining and analyzed the EVI1-expression in glioma tissue. To determine the relationship between *EVI1* and *EGFR*, we induced siRNA-mediated knockdown of *EVI1* in GBM cell lines. To investigate the region that was essential for the EVI1 regulation of *EGFR* expression, we conducted promoter reporter assays. We performed WST-8 assay to investigate whether EVI1 affected on the proliferation of GBM cells or not.

**Results:**

It was observed that 22% of GBM tissues had over 33% of tumor cells expressing EVI1, whereas no lower-grade glioma tissue had over 33% by immunohistochemistry. In A172 and YKG1 cells, the expression levels of EGFR and EVI1 correlated. Analysis of the *EGFR* promoter region revealed that the EGFR promoter (from − 377 to − 266 bp) was essential for the EVI regulation of *EGFR* expression. We showed that EVI1 influenced the proliferation of A172 and YKG1 cells.

**Conclusion:**

This is the first study reporting the regulation of *EGFR* transcription by EVI1 in GBM cells.

**Electronic supplementary material:**

The online version of this article (10.1007/s11060-019-03310-z) contains supplementary material, which is available to authorized users.

## Introduction

In 1988, ecotropic viral integration site-1 (*EVI1*) was originally identified as a common site of retroviral integration in murine myeloid tumors [1, 2]. The human *EVI1* is located on chromosome 3q26 and produces a transcription factor that contains two DNA-binding zinc finger domains: one binds to a GATA-like consensus motif and the other binds to a v-ets erythroblastosis virus E26 oncogene homolog (ETS)-like motif [3, 4]. While regulating target gene expression, EVI1 interacts with transcription coregulators such as the C-terminal binding domain (CtBP), cAMP-responsive element-binding protein-binding protein (CBP), and p300/CBP-associated factor (P/CAF) [5]. EVI1 represses transforming growth factor-beta (TGF-β) signaling and activates PI3K/Akt/mTOR signaling [6, 7]. Elevated EVI1 expression is an unfavorable prognostic factor in human acute myeloid leukemia and some solid cancers [8–12].

EVI1 is closely related to embryonic neural development. In one study, the EVI1 homozygous mutant mouse embryos that died at 10.5 day post-coitus had a defect in the cranial ganglia and developed failure of the peripheral nervous system [13]. EVI1 is related to the Notch signaling pathway, which is important for cell-fate specification in a developing mammalian nervous system [5, 6]. This evidence showed that EVI1 is a key molecule in neurogenesis. Further, EVI1 has been reported to contribute to worsening of glial tumors [7, 8], suggesting that it might have an oncogenic role in glioma genesis. However, studies aiming to investigate EVI1 function in glioma have been few.

Recently, through cDNA microarray Gene Chips experiments, Chapeau et al. revealed that EVI1 regulated 621 cancer-associated genes in Hela and SKOV3 cells [9]. Among these EVI1 target genes, we were interested in *epidermal growth factor receptor* (*EGFR*). Signal transduction via the tyrosine kinase receptor EGFR contributes to cell proliferation, apoptosis inhibition, and angiogenesis in cancer cells. *EGFR* is the most important downstream target gene of the Notch signaling pathway [10, 11]. Since amplification and/or mutations in *EGFR* represent a genetic abnormality in primary glioblastoma multiforme (GBM), *EGFR* has been regarded as a pivotal target for GBM studies.

Therefore, we presumed that EVI1 might have a significant role in GBM by regulating *EGFR* gene expression.

## Materials and methods

### Microarray data

We downloaded the four GEO series (GSE2223, GSE4271, GSE23806, and GSE43378) from the GEO database in NCBI (https://www.ncbi.nlm.nih.gov/geo/). Here we chose 29 GBM samples, except gliosarcoma, from GSE2223. In the same way, we chose 76 GBM samples from GSE4271, chose 32 conventional GBM cell line samples from GSE23806 and chose 32 GBM samples from GSE43378. We used pairs of probe-sets of MECOM and EGFR to evaluate the corresponding expression levels, demonstrated by scatter plot. (Supplementary Fig. 1).

### Sample collection

This study was approved by the Ethics Committee of the University of Miyazaki Hospital, Miyazaki, Japan. All patients and their families gave informed consent for the use of the resected tissues. In this study, we included patients who had infiltrative glioma with histological grades II and III (lower-grade glioma, n = 27; 15 men and 12 women; median age, 41 years) or grade IV (GBM, n = 37; 27 men and 10 women; median age, 67 years). We used eight normal brain tissues as normal controls. Two or more pathologists examined all tumor tissue sections according to the WHO criteria. The tissue samples were obtained from tumors resected at the Department of Neurosurgery, University of Miyazaki Hospital between May 2014 and January 2017.

### Immunohistochemical staining and evaluation of the paraffin-embedded tissues

To evaluate the expression of EVI1 using immunohistochemistry, we used the diluted EVI1 antibody (1:500, Cell Signaling Technology, #2593). To evaluate the expression of EGFR using immunohistochemistry, we used the diluted EGFR antibody (1:200, Leica microsystems, NCL-EGFR). The primary antibody was detected using the Dako EnVision™ + system—HRP Labeled Polymer (anti-rabbit) (Dako, K4002) and the Dako EnVision™ + system–HRP Labeled Polymer (anti-mouse) (Dako, K4000). The slides were counterstained using Meyer’s hematoxylin. Without knowledge on the clinical information, we photographed three regions of high cellularity in each tissue slide using a BZ-9000 microscope (Keyence, Osaka, Japan). The numbers of both EVI1-positive cells and cells in total were calculated using BZ-II Analyzer version 1.42 and BZ-II Dynamic Cell Count version 1.01 (Keyence). The mean values were defined as the EVI1-positive cell rate. The scores of the EVI1-positive tumor cells were defined as (−) if less than 5% (+) if 5–33% (++) if 33–50%, and (+++) if more than 50%. The threshold values were defined based on those described in the previous study by Hou et al. [8]

### PCR and real-time quantitative PCR

Total RNA was isolated using the RNeasy Mini Kit, according to the manufacturer’s instructions (Qiagen, No.74104). Total RNA in each sample was reverse transcribed using the Super Script VILO cDNA synthesis kit (Invitrogen, 11754-050). Quantitative PCR was performed on the StepOnePlus (Applied bio systems) using THUNDERBIRD SYBR qPCR Mix (TOYOBO, QPS- 201). The primers used in this study are as described in Supplementary Fig. 2.

### EVI1 RNA interference

The final RNA concentration was 16.6 nM. The siRNA sequences targeting human EVI1 were as described in Supplementary Fig. 2 [12]. The universal negative control siRNA was purchased from NIPPON GENE (NIPPON GENE CO. Ltd. Toyama, Japan).

### Western blotting

We used the primary antibody (EGFR, Cell Signaling Technology_#4267_1:1000 and b-Actin, SIGMA-ALDRICH_A5441_1:5000). The secondary antibody was applied and the signal was visualized on ImageQuant LAS 4000 (GE Healthcare) using the Lumi-Light plus Western Blotting Substrate (Roche, 12015196001).

### Constructs

All constructs that we used in this study are described in details in Electrical Supplementary Material 1.

### Luciferase assay

We performed the dual luciferase assays according to the manufacturer’s instructions (Promega, E1910).

### Cell proliferation assay

Cell proliferation assays were performed with a Cell Counting Kit-8 (Dojindo, Japan) according to the manufacturer’s protocol.

### Statistical analysis

**Fig. 1:**
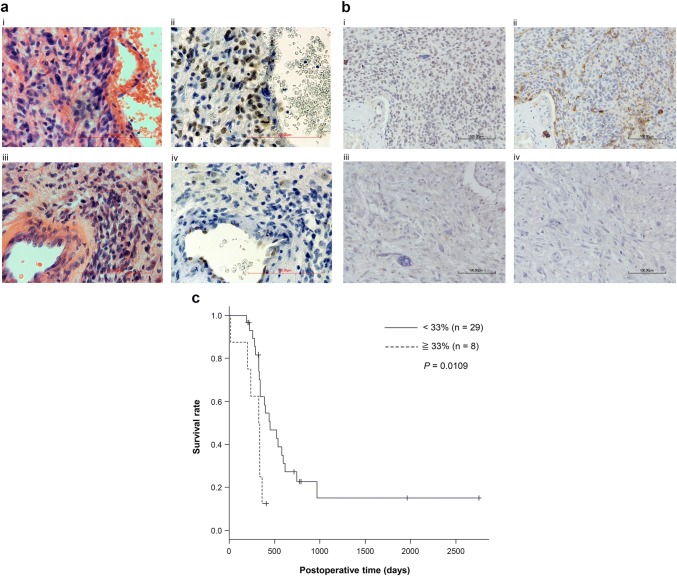
Immunohistochemical staining for EVI1 in glioma tissue. **a** Representative HE staining (× 400) (i, iii) and EVI1 immunohistochemistry images (ii, iv) of GBM tissues; (i) and (ii) are GBM tissues expressing high levels of EVI1, whereas (iii) and (iv) are GBM tissues expressing low levels of EVI1. The scale bars represent 100 μm. **b** Representative immunohistochemistry images (× 200) of GBM tissues; (i) and (ii) are GBM tissues expressing high levels of EVI1, whereas (iii) and (iv) are GBM tissues expressing low levels of EVI1. (i, iii) were EVI1 immunohistochemistry, whereas (ii, iv) were EGFR immunohistochemistry. The scale bars represent 100 μm. **c** Log-rank test for the survival curves stratified by EVI1 expression in 37 GBM tissues shows a worse overall survival rate in patients with high EVI1 expression (EVI1-positive cell rate of ≥ 33%; *n* = 8) than in those with low EVI1 expression (EVI1-positive cell rate of < 33%; *n* = 29) (*P* = 0.0109)

**Fig. 2:**
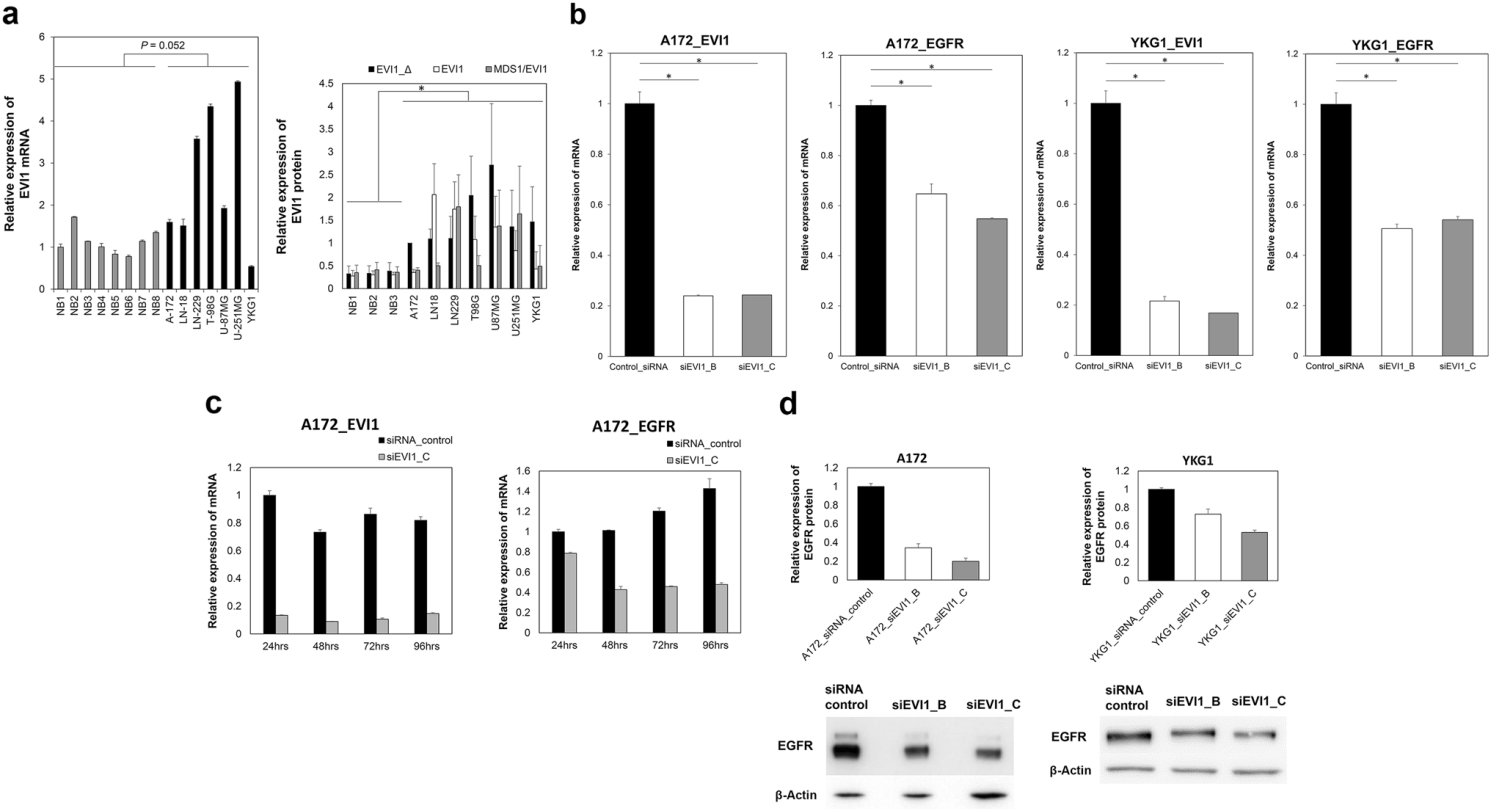
**a** The expression levels of *EVI1* in GBM cell lines. The mRNA expressions of *EVI1* (left panel) and protein of EVI1 (right panel) in 7 GBM cell lines (A-172, LN-18, LN-229, T98G, U-87MG, U-251MG, and YKG1) are shown. In the western blot analysis of EVI1 in GBM cell lines and normal brain tissues, the beta-actin was used as the loading control. Triplicates were analyzed and results were presented as changes in fold, relative to the A172-EVI1 expression level. The error bars represent the standard deviation. (*P* < 0.05) (The representative data was shown in Supplementary Fig. 4) The graphs show the integrated density measured by Image J and normalized to beta-actin. **b** siRNA-mediated *EVI1* knockdown and the expression level of *EGFR* in GBM cells. (A172, YKG1) The mRNA expressions of *EVI1* and *EGFR* were normalized to *18S* mRNA (left panel, A172 and right panel, YKG1). The black bars represent negative control, the white bars represent EVI1 knock down (siEVI1_B) and the gray bars represent EVI1 knock down (siEVI1_C). All assays were performed in triplicate. The results were presented as changes in fold, relative to the negative control. Quantitative PCR data were presented as mean ± SD mRNA expression, *P < 0.05. **c** The relative mRNA expressions of *EVI1* (left panel) and *EGFR* (right panel) in the A172 cells that underwent siRNA transfection. *EGFR*-mRNA levels decreased from 24 to 96 h. The highest difference in *EGFR*-mRNA expression level relative to the negative control was observed at 96 h after transfection. **d** Western blot analysis of EGFR in GBM cells (left panel, A172 and right panel, YKG-1). The beta-actin was the loading control. Triplicates were analyzed and results were presented as changes in fold, relative to the negative control. The graphs show the integrated density measured by Image J and normalized to beta-actin. The error bars represent the standard deviation. (*P* < 0.02)

**Fig. 3:**
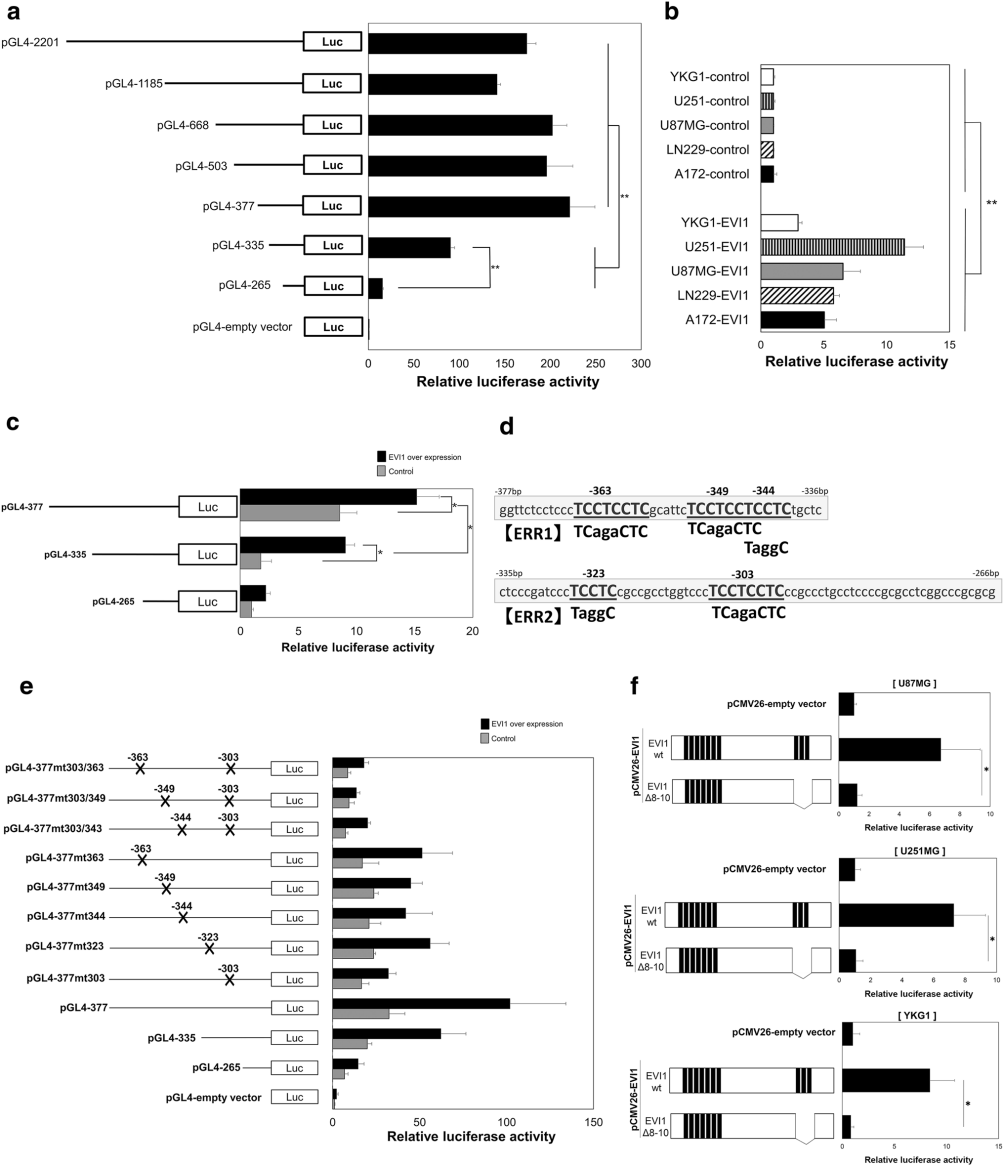
The importance of ERR for EVI1 regulation of *EGFR* transcription. All dual luciferase assays in the GBM cells were performed in triplicate. The values and error bars depict the mean values ± SD. **a** Various length fragments of the *EGFR* promoter region were inserted upstream of the luciferase gene in the reporter plasmid pGL4.10 [*luc2*]. The relative luciferase activity was normalized after transfection in U-87MG. A pGL4.10 [*luc2*] plasmid was used as control (pGL4-empty vector). **P < 0.02. **b** The relative luciferase activities after transfection of the pGL4-377 plasmid with the EVI1 overexpression vector (pCMV26-EVI1) or control vector (pCMV26-control). The overexpression of EVI1 protein in GBM cells (U87MG, YKG1) was validated by western blot analysis (Supplementary Fig. 7). This experiment was performed using the GBM cell lines LN229, YKG-1, U-251MG, U-87MG, and A-172, **P < 0.02. **c** The relative luciferase activities in U-87MG after transfection of the luciferase reporter plasmids, with various lengths of *EGFR* promoter fragments integrated. Each plasmid was transfected with the EVI1 overexpression vector (pMXs-EVI1; black bars) or the control vector (pMXs-control; gray bars). pGL265 with the control vector was used as control. All luciferase reporter assays were performed in quadruplicate. The values and error bars depict the mean ± SD, *P < 0.05. **d** The panel shows the sequences of ERR (EVI1 regulatory region). The sequences are numbered according to their positions, relative to the site of translation start (+ 1 at ATG). The two boxed sequences represent ERR1 (− 377 bp to − 336 bp) and ERR2 (− 335 bp to − 266 bp) sites. The mutation regions that we used in the *EGFR* promoter mutant luciferase assay are shown in this schema. **e** The *EGFR* promoter regions (− 377 bp to − 63 bp upstream of the translational starting site), which contain mutations (X) in the putative EVI1 binding sites, are inserted upstream of the luciferase gene in the reporter plasmid pGL4.10 [*luc2*]. The numbers placed on the X mark represent a region from the site of the translational start of the *EGFR* promoter. Each plasmid is transfected with the EVI1 overexpression vector (pMXs-EVI1, black bars) or the control vector (pMXs-control, gray bars) into U-87MG. **P < 0.02. **f** Enhancement of the promoter activity of EGFR by EVI1 via the C-terminal DNA-binding domain. The structures of the wild-type EVI1 (EVI1 wt) and the mutant EVI1 containing a deletion of the C-terminal DNA-binding domains (EVI1 Δ8-10) are depicted. U-87MG, U-251MG, and YKG-1 cells were co-transfected with the *EGFR* reporter vector (pGL4-377), pRL-TK, and each EVI1 overexpression vector (pCMV26-EVI1/ pCMV26-EVI1Δ8-10). pCMV26-control was used as control. All luciferase reporter assays were performed in triplicate. The values and error bars depict the mean ± SD, *P < 0.05

**Fig. 4:**
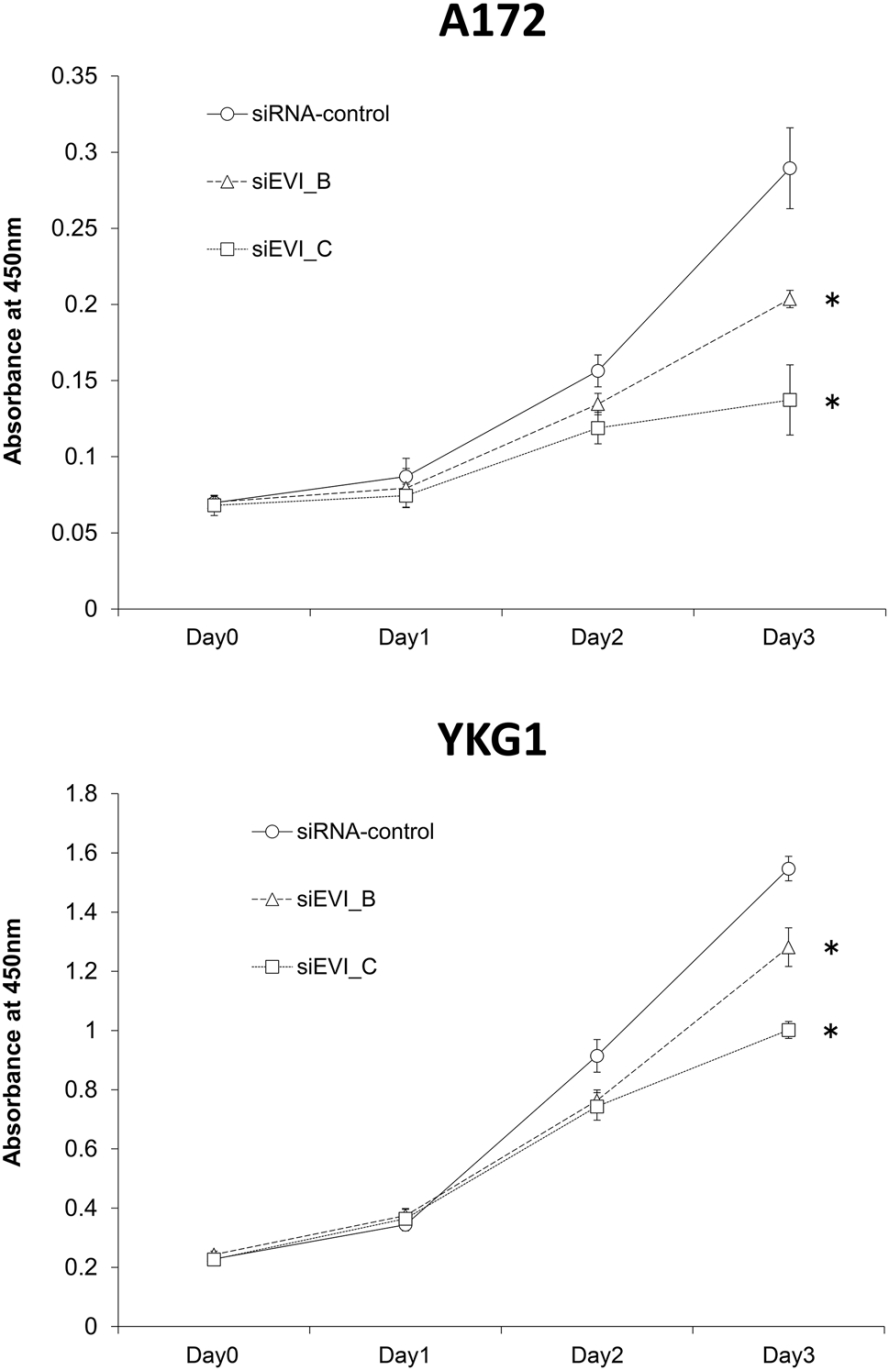
The cell proliferation curves of the A172 and YKG-1 cells transduced with siRNA against EVI1 (siEVI1_B and siEVI1_C), compared with those of the negative control (siRNA-control). On day 0, the cells were transfected with siRNA. Cell counting was carried out at 24, 48, and 72 h after siRNA transfection. Data shown are the mean ± SD of the quadruplicate analyses. Statistical significance was determined by the Student’s *t* test, *P < 0.05

The correlations between the expression levels of EVI1 and EGFR (GSE2223, GSE4271, GSE23806, and GSE43378) were calculated using Spearman’s rank correlation coefficient (Supplementary Fig. 1). The difference in the survival curves was analyzed by the log-rank test (Fig. 1c). We compared the *EVI1*-mRNA levels of the target genes between the GBM cell lines and the normal brain tissues using Mann–Whitney *U-*test (Fig. 2a). All statistical analyses were performed using the Student’s *t* test (Figs. 2, 3, 4). We used EasyR software for statistical analysis. *P* values < 0.05 were considered significant.

## Results

### Positive correlation between the expression levels of EVI1 and EGFR in GBM

To investigate whether EVI1 was associated with EGFR expression in GBM, we referred to GEO data and calculated the correlation coefficients of the expression levels of EVI1 and EGFR. A positive correlation was found between EVI1 and EGFR expressions in each of the GBM series, including GSE2223 (*r* = 0.429–0.51); GSE4271 (*r* = 0.379–0.462); GSE23806 (*r* = 0.445–0.606); and GSE43378 (*r* = 0.461–0.551) (Supplementary Fig. 1).

### The EVI1 expression level was relatively high in GBM tissue and might contribute to worse prognosis

To clarify the role of EVI1 in glioma genesis, we performed immunohistochemical staining and analyzed the EVI1-positive cell rates in 37 GBM and 27 lower-grade glioma (LGG) tissue samples. EVI1 was detected in the nuclei of the glioma cells, and EVI1-positive tumor cells were observed diffusely in glioma tissue. We observed the heterogeneity of nuclei at the point of staining intensity (Fig. 1a). In addition, the cases those were stained well by EVI1 were also stained well by EGFR (Fig. 1b).

On examination of the 37 GBM tissue samples, the positive rate was 0–5% in 15 cases, 5–33% in 14 cases, 33–50% in six cases, and over 50% in two cases. On the other hand, among the 27 LGG tissue samples, the positive rate was 0–5% in 13 cases and 5–33% in 14 cases; none of the cases had over 33% positive rate (Table 1). Because no LGG tissues had over 33% of tumor cells expressing EVI1, we chose the 33% as the cut-off level in the survival curve. Of the 37 GBM cases, eight cases that had over 33% EVI1-positive rate showed significantly worse prognosis (*P* = 0.0109). The one-year survival rate was 12.5% for the cases with over 33% positive rate and 62.2% for the cases with 0–33% positive rate (Fig. 1c). On the other hand, we examined the relation between the level of *EGFR*-mRNA expression and post-operative days in these patients with GBM. However, the result showed no significant difference (Supplementary Fig. 3).

**Table 1:** EVI1-positive cell rates in the GBM and LGG tissues

	LGG	GBM
−	13	15
+	14	14
++	0	6
+++	0	2
Total	27	37

Among the 37 GBM tissue samples, the positive rate was over 33% in eight cases. However, among the 27 LGG tissue samples, none showed a positive rate of over 33%. The scores of the EVI1-positive tumor cells were defined as (−) if less than 5% (+) if 5% to 33% (++) if 33% to 50%, and (+++) if more than 50%.

### The EVI1 expression levels were more varied in the GBM cell lines than in the normal brain tissue samples

To investigate the expression levels of *EVI1* in various GBM cell lines, we performed quantitative PCR and western blotting using seven GBM cell lines (i.e., A-172, LN-18, LN-229, T98G, U-87MG, U-251MG, and YKG-1) and compared them with those in normal brain tissues (i.e., NB-1, NB-2, NB-3, NB-4, NB-5, NB-6, NB-7, and NB-8). The levels of *EVI1*-mRNA in the GBM cell lines varied and showed no significant difference; (*P* = 0.052) (Fig. 2a, left panel), however, the protein levels of EVI1 in GBM cell lines were higher compared with those in normal brain tissues (*P* < 0.05) (Fig. 2a, right panel, The representative data was shown in Supplementary Fig. 4).

### siRNA-mediated EVI1 knockdown decreased the expression levels of EGFR

To determine the regulatory mechanism between *EVI1* and *EGFR*, we induced knockdown of *EVI1* in GBM cells because EVI1 is a transcription factor. First, we induced siRNA-mediated knock down in A172, LN18, LN229, T98G, U87MG, U251MG, and YKG1. As a result, the *EVI1*-mRNA levels in these GBM cell lines were reduced. In addition, we observed reduction in *EGFR*-mRNA and EGFR protein expression in response to the decrease in *EVI1* mRNA (Supplementary Figs. 5, 6). Among these GBM cell lines, the differences in *EGFR*-mRNA between knock down group and control group were bigger in A172 and YKG1 than other GBM cell lines. Thus, we chose these two cell lines for siRNA-mediated EVI1 knock down experiments.

Transfection with the siRNAs achieved > 75% decrease in *EVI1* mRNA expression in the A172 and YKG-1 cells. In the A172 cells, *EGFR*-mRNA expression decreased by 35% and 45% after transfection with siEVI1_B and siEVI1_C, respectively, whereas in the YKG1 cells, the expression decreased by 49% and 45% after transfection with siEVI1_B and siEVI1_C, respectively (Fig. 2b).

In the A172 cells transfected with siEVI1_C, reduced *EVI1* and *EGFR*-mRNA levels were observed at 24, 48, 72, and 96 h; the largest difference in the *EGFR*-mRNA expression level, relative to the negative control, was observed at 96 h after transfection (66% reduction) (Fig. 2c). Therefore, we conducted western blot assay 96 h after transfection and found that the *EGFR* protein expression in GBM cells decreased after treatment with the siRNAs against *EVI1*, compared with the effects in the negative control. In the A172 cells, *EGFR* protein expression decreased by 65% and 79% after transfection with siEVI1_B and siEVI1_C, respectively, whereas in the YKG1 cells, the expression decreased by 27% and 47% after transfection with siEVI1_B and siEVI1_C, respectively (Fig. 2d).

### The importance of the EVI1 regulatory region in the EVI1 regulation of EGFR transcription

To clarify the molecular mechanisms underlying the transcriptional regulation of *EGFR* by EVI1, we attempted to determine the essential promoter region of *EGFR* using luciferase assay. In the luciferase assays, we mainly used U87MG cells because these cells received few cell damages by the HilyMax compared to other GBM cell lines. The luciferase activity reduced significantly with pGL4-335 (59%) and pGL-265 (93%) than with pGL-377 (Fig. 3a). This result suggested that *EGFR* transcription was primarily driven by DNA derived from the 112 bp fragment, which was located − 377 bp to − 266 bp upstream of the *EGFR* promoter. When pGL4-377 was co-transfected with the *EVI1* overexpression vector into five GBM cell lines (A172, LN229, U-87MG, U-251MG, and YKG-1), exogenous *EVI1* enhanced the luciferase activities by 2.9-fold in YKG1 and by 11.4-fold in U-251MG (Fig. 3b). Thus, EVI1 had a positive influence on *EGFR* transcription in GBM cells. To determine the more important region (− 377 bp to − 336 bp vs. − 335 bp to − 266 bp) for the EVI1 regulation of *EGFR* transcription, luciferase activities were measured after co-transfection of the pGL4-265, pGL4-335, and pGL4-377 plasmids with the EVI1 overexpression vector (pMXs-EVI1) into the U-87MG cells. The luciferase activities were enhanced by exogenous EVI1 in both the pGL4-335 and pGL4-377 plasmids. Furthermore, the luciferase activity was significantly higher in pGL4-377 than in pGL4-335 (Fig. 3c). The results suggested that both *EGFR* promoter regions were important for the EVI1 regulation of *EGFR* transcription. We designated the promoter region in − 377 bp to − 266 bp as the EVI1 regulatory region (ERR). In ERR, − 377 bp to − 336 bp were designated as ERR1 and − 335 bp to − 266 bp were designated as ERR2 (Fig. 3d).

### The role of the polypyrimidine stretches in the EVI1 regulatory region in the EVI1 activation of the EGFR promoter activity

We focused on the EVI1-binding sequences in ERR. EVI1 contains two independent DNA-binding domains, including the N-terminal and C-terminal domains. Previous reports showed that the EVI1 N-terminal domains recognized a GATA-like motif and the C-terminal domains recognized an ETS-like motif [3, 13]. Notably, ERR had no GATA-like motif, but it was rich in polypyrimidine stretches. We presumed that EVI1 needs at least two regions of ERR1 and ERR2 to regulate *EGFR* transcription. Furthermore, previous studies have shown that two regions of TC-rich direct-repeat sequences (general motif; TCCTCCTCC) in the *EGFR* promoter are essential for the regulation of *EGFR* transcription [14, 15]. Therefore, we generated eight *EGFR* mutant promoter constructs, viz., pGL4-377mt303, pGL4-377mt323, pGL4-377mt344, pGL4-377mt349, pGL4-377mt363, pGL4-377mt303/343, pGL4-377mt303/349, and pGL4-377mt303/363. We transfected these eight plasmids with the EVI1 overexpression vector into U-87 MG cells. As a result, the mutant plasmid had reduced *EGFR* transcriptional activity. In particular, transfection with pGL4-377mt303/343, pGL4-377mt303/349, and pGL4-377mt303/363 completely abolished EVI1 efficacy to enhance EGFR promoter activity (Fig. 3e).

### * The promoter activity of EGFR is enhanced by EVI1 *via* the C-terminal DNA-binding domain*

The EVI1 mutant construct with deletion of the C-terminal DNA-binding domain (pCMV26-EVI1Δ8-10) was transfected with pGL4-377 into U-87MG, U-251MG, and YKG1 cells (Fig. 3f). *EGFR* promoter activity was enhanced in the presence of transfected wild-type EVI1 but markedly reduced after transfection of the EVI1 deletion mutant construct (pCMV26-EVI1Δ8-10). This suggested that the loss of the pCMV26-EVI1Δ8-10 transcriptional enhancer activity could be attributed to losing the capacity to bind to the *EGFR* promoter region.

### EVI1 knockdown suppressed the proliferation of GBM cells

We investigated whether *EVI1* knockdown affected the proliferation of A172 and YKG-1 cells. On day 3, the transfection of siRNAs against EVI1 significantly suppressed the proliferation of both cell types compared with the effects in the negative control (Fig. 4).

## Discussion

In this study, we introduced the high tissue expression of EVI1 in patients with GBM might relate to poor prognosis. Next, we demonstrated that EVI1 regulated *EGFR* transcription and affected the proliferation of GBM cells. Further, we found that both TC-rich stretches in the *EGFR* promoter were essential for the EVI1 regulation of *EGFR* transcription. Only few reports on glioma focused on EVI1. In this study, we first reported that EVI1 regulated *EGFR* transcription in GBM cells.

*EGFR* amplification occurs in about 50% of primary GBM [16]. To date, several transcription factors have been reported to contribute to the regulation of *EGFR* transcription [14, 15, 17, 18]. Among these reports, the one by Johnson et al. is intriguing [14, 19] because they identified the two pairs of direct-repeat sequences, which conformed to the general motif TCCTCCTCC, on the *EGFR* promoter. We reported in this study that EVI1 also needed the TC-rich stretches in ERR, and the ability of EVI1 to regulate *EGFR* transcription depends on both ERR1 and ERR2 sites.

According to a previous study, the relations between *EGFR* expression and the prognosis of patients with GBM are controversial [20]. In this study, the result showed no significant relation between the *EGFR*-mRNA expression levels and the post-operative days (Supplementary Fig. 3). This result suggested that high-EVI1 expression could be correlated with worse prognosis of GBM depend on another mechanism unrelated to *EGFR* regulation.

According to the cBioPortal for cancer genomics data (https://www.cbioportal.org/index.do), the frequency of *EVI1* genetic alteration was lower in glioma (2.1% of 283 cases) than in other solid cancers (lung squamous cell carcinoma, 44.1%; ovarian serous cyst adenoma, 33.8%; esophageal carcinoma, 25.5%; and neuroendocrine prostate cancer, 22.4%). Therefore, most researchers on glial tumors had less interest in this transcription factor. However, we showed that in patients with glioma, EVI1 might be related to poor prognosis.

There were some limitations to this report. First, our study population was relatively small and the design was retrospective. Second, our findings were only based on in vitro assays. To validate the role of EVI1 in GBM, in vivo assays are critical. Nevertheless, we showed the exciting potential of EVI1 to interrupt glioma cell proliferation. Therefore, further studies should clarify the role of EVI1 in patients with glioma.

## Conclusion

We reported in this study that EVI1 regulated *EGFR* transcription and effect on the cell proliferation in GBM cells. The TC-rich stretches in the *EGFR* promoter (from − 377 to − 266 bp, relative to the EGFR translational start site) was essential for the EVI regulation of *EGFR* expression.

## Electronic supplementary material

Below is the link to the electronic supplementary material.

Supplementary file1 (DOCX 24 kb)

Supplementary file1 (TIFF 1520 kb)

Supplementary file1 (TIFF 459 kb)

Supplementary file1 (TIFF 250 kb)

Supplementary file1 (TIFF 297 kb)

Supplementary file1 (TIFF 184 kb)

Supplementary file1 (TIFF 271 kb)

Supplementary file1 (TIFF 645 kb)
