# Supplementary material for: Ecotropic viral integration site 1 regulates EGFR transcription in glioblastoma cells
Source: J Neurooncol. 2019 Oct 15;145(2):223–31. doi: 10.1007/s11060-019-03310-z (PMC6856030; doi:10.1007/s11060-019-03310-z)
Supplement: Supplementary file 1 — Supplementary file1 (DOCX 24 kb) [file 11060_2019_3310_MOESM1_ESM.docx]

**Ecotropic viral integration site 1 regulates EGFR transcription in glioblastoma cells**
*Journal of Neuro-Oncology*

Asako Mizuguchia*, Shinji Yamashitaa, Kiyotaka Yokogamia, Kazuhiro Morishitab, Hideo Takeshimaa
*Corresponding author: Asako Mizuguchi: Assistant Professor of Department of Neurosurgery, Faculty of Medicine, University of Miyazaki, Miyazaki, Japan

Tel: +81-985-85-3128, Fax: +81-985-84-4571

E-mail address: asako_mizuguchi@med.miyazaki-u.ac.jp

Constructs

EVI1 overexpression vectors

We used two kinds of vectors to constitutively express the active human EVI1. One vector (pMXs-EVI1) was generated by cloning a PCR-amplified *EVI1* fragment into the BamHI/XhoI restriction sites of pMXs-IRES-GFP_Retroviral_Expression_Vector (pMXs-control, Cell BIOLABS) in-frame. Another vector (pCMV26-EVI1) was generated by cloning a PCR-amplified *EVI1* fragment into p3×FLAG-Myc-CMV26^TM^ Expression Vector (pCMV26-control, SIGMA-ALDRICH), using EcoRI/ Bam HI restriction sites[1].

Luciferase reporter constructs containing the EGFR promoter

The luciferase reporter constructs containing the EGFR promoter, including pGL4-2201, pGL4-1185, pGL4-668, pGL4-503, pGL4-377, pGL4-335, and pGL4-265, were prepared by cloning the *EGFR* promoter region of the genomic DNA into the pGL4.10[*luc2*] vector (pGL4-empty, Promega, Madison, WI), using the Kpn I/EcoR V sites. The 3′-end of each of the following EGFR–luciferase constructs was at -64 bp, relative to the *EGFR* translational start site, whereas the 5′-ends were mapped to the following positions: pGL4-2201 (-2201 bp), pGL4-1185 (-1185 bp), pGL4-668 (-66 8bp), pGL4-503 (-503 bp), pGL4-377(-377 bp), pGL4-335 (-335 bp), and pGL4-265(-265 bp). For the construction of the *EGFR* promoter mutant luciferase constructs, we replaced the TCCTCCTC with the TCagaCTC variation (-303 bp, -349 bp, -363 bp) or the TCCTC with TaggC variation (-344 bp, -323 bp), using the Q5 site-directed mutagenesis kit (BioLabs, E0554S). The pGL4-377mt303 was mutated at the site of -303 bp, upstream from the EGFR translation start site. In the same manner, we prepared pGL4-377mt323 (-323 bp), pGL4-377mt344 (-344 bp), pGL4-377mt349 (-349 bp), pGL4-377mt363 (-363 bp), pGL4-377mt303/343(-303 bp and -343 bp), pGL4-377mt303/349 (-303 bp and -349 bp), and pGL4-377mt303/363 (-303 bp and -363 bp). Afterward, the *EGFR* promoter sequence was verified by sequencing.

EVI1 deletion mutant constructs

The pCMV26-EVI1Δ8-10 construct, an EVI1 mutant construct with deletion of the C-terminal DNA-binding domain vector, was kindly provided by Dr. Kurokawa of the University of Tokyo [2].

[**1**] Morishita K, Parganas E, Douglass EC, Ihle JN. Unique expression of the human Evi-1 gene in an endometrial carcinoma cell line: sequence of cDNAs and structure of alternatively spliced transcripts. *Oncogene.* 1990; 5(7):963-971.

[2] Kurokawa M, Mitani K, Irie K, et al. The oncoprotein Evi-1 represses TGF-beta signalling by inhibiting Smad3. *Nature.* 1998; 394(6688):92-96.
