# Supplementary figures and images for: Ecotropic viral integration site 1 regulates EGFR transcription in glioblastoma cells
Source: J Neurooncol. 2019 Oct 15;145(2):223–31. doi: 10.1007/s11060-019-03310-z (PMC6856030; doi:10.1007/s11060-019-03310-z)

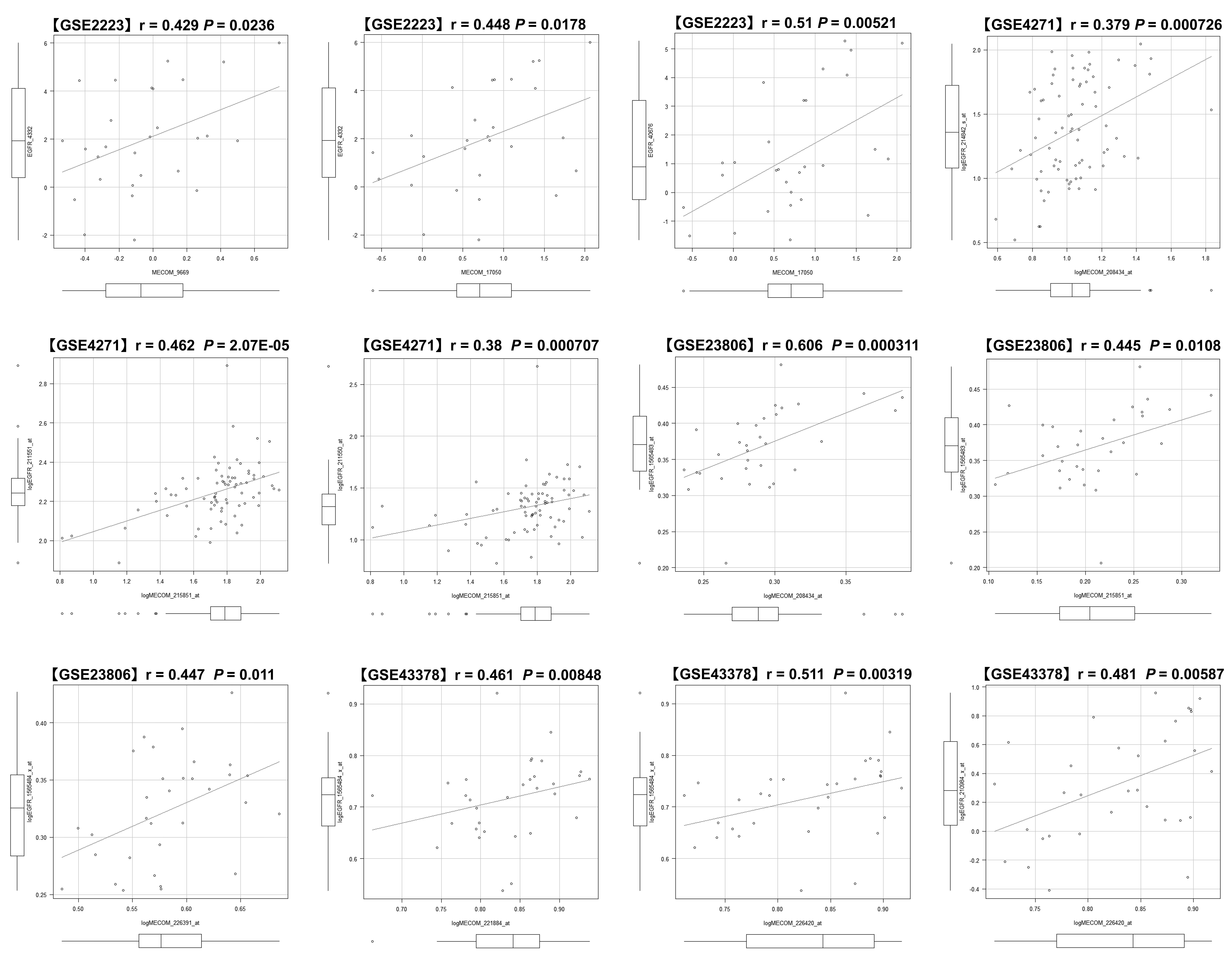

Supplement: Supplementary file 2 — Supplementary file1 (TIFF 1520 kb) [file 11060_2019_3310_MOESM2_ESM.tif]

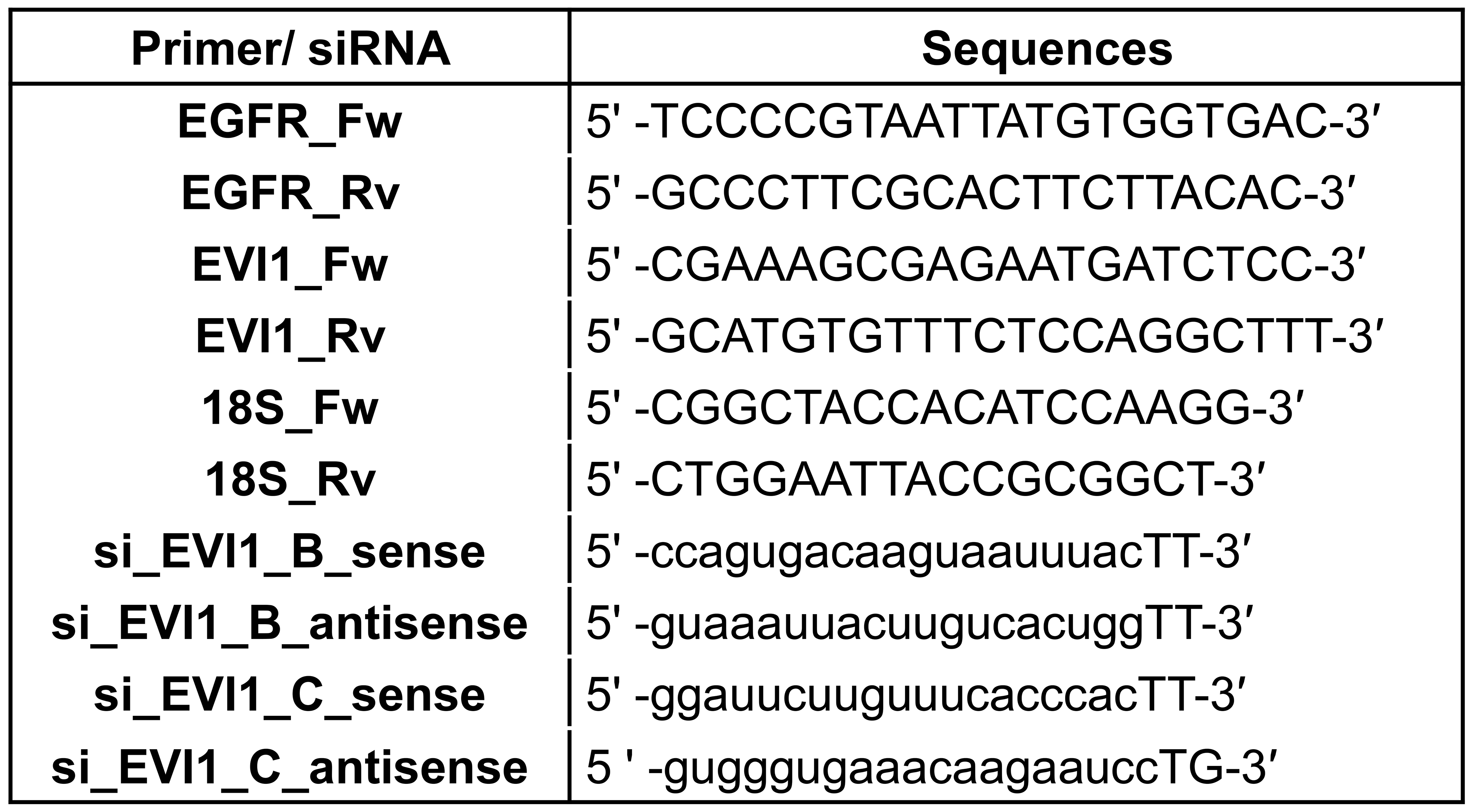

Supplement: Supplementary file 3 — Supplementary file1 (TIFF 459 kb) [file 11060_2019_3310_MOESM3_ESM.tif]

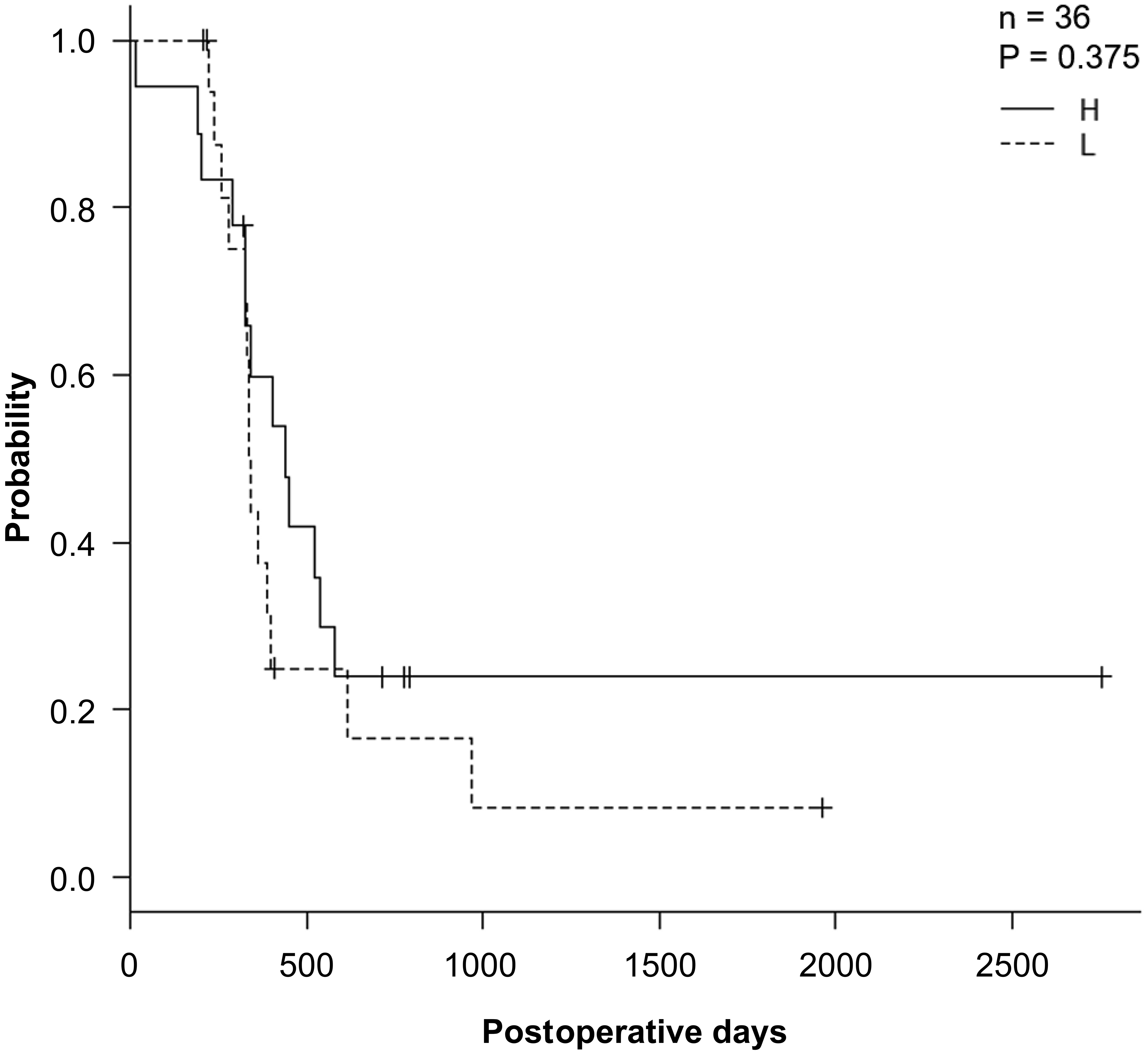

Supplement: Supplementary file 4 — Supplementary file1 (TIFF 250 kb) [file 11060_2019_3310_MOESM4_ESM.tif]

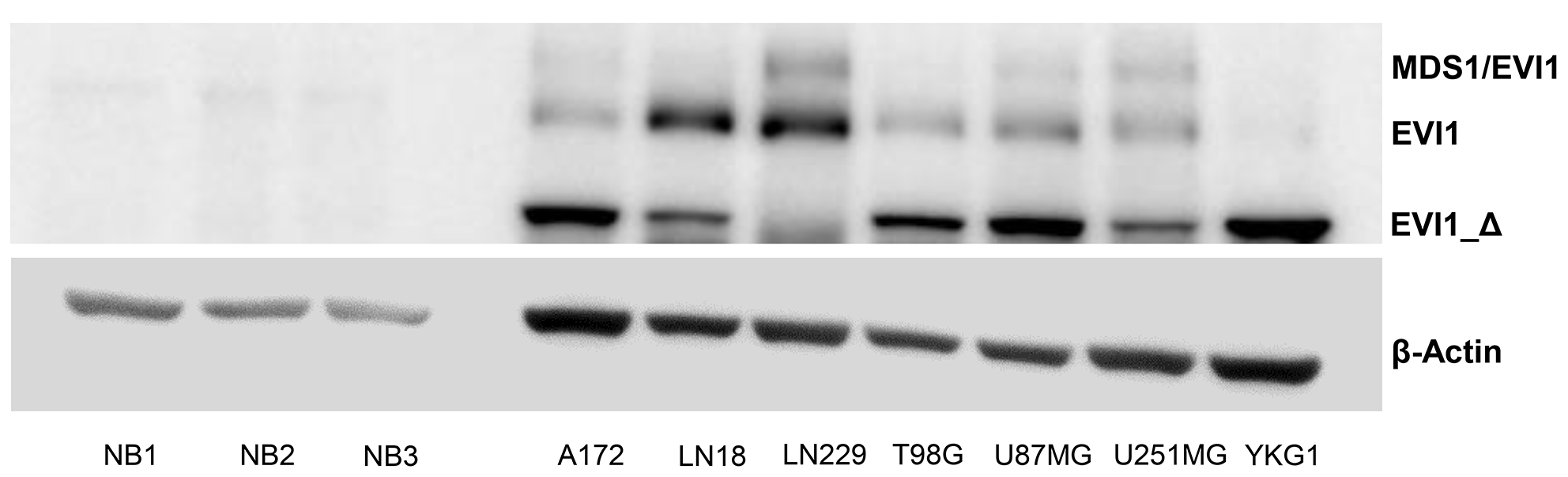

Supplement: Supplementary file 5 — Supplementary file1 (TIFF 297 kb) [file 11060_2019_3310_MOESM5_ESM.tif]

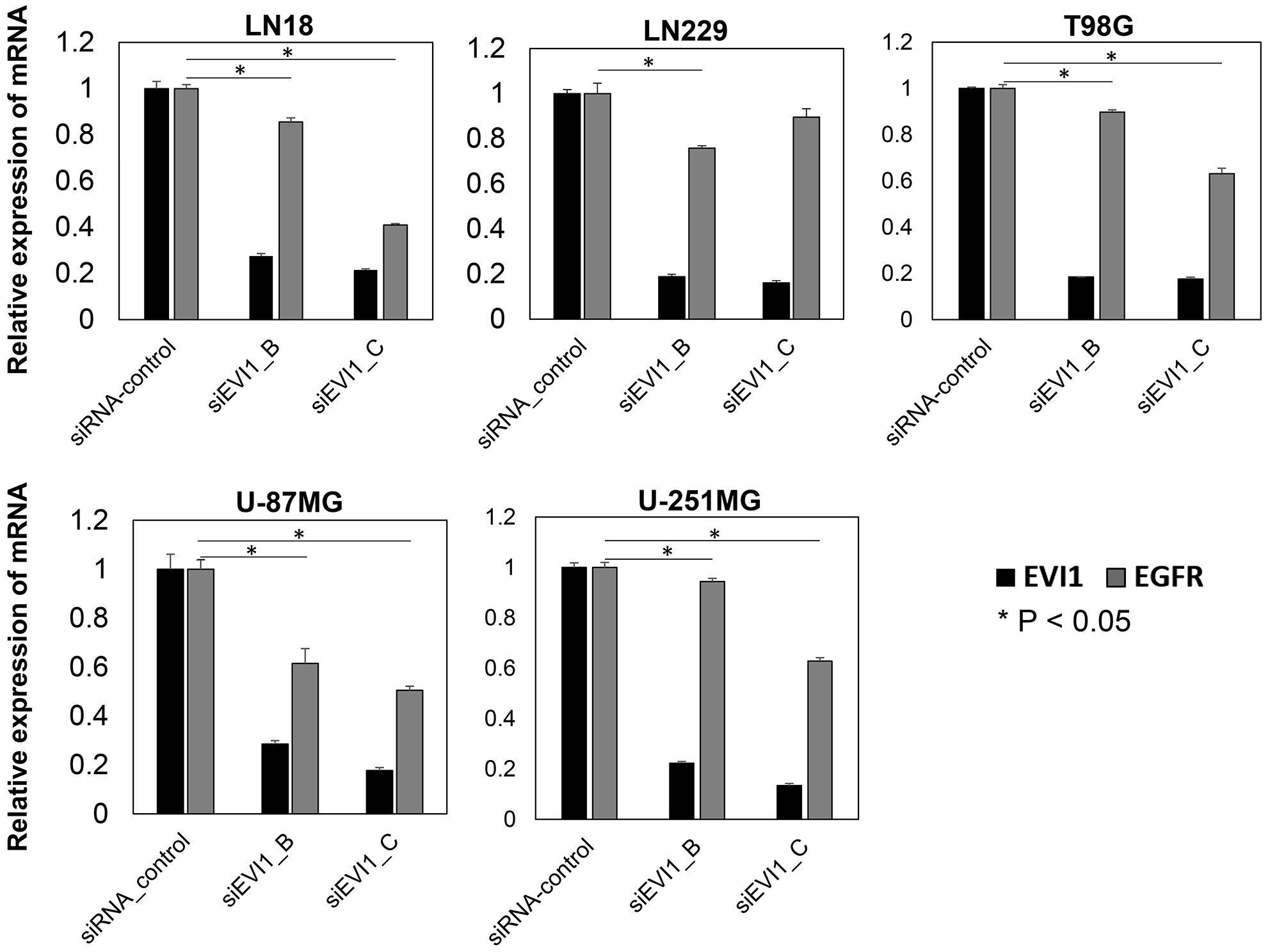

Supplement: Supplementary file 6 — Supplementary file1 (TIFF 184 kb) [file 11060_2019_3310_MOESM6_ESM.tif]

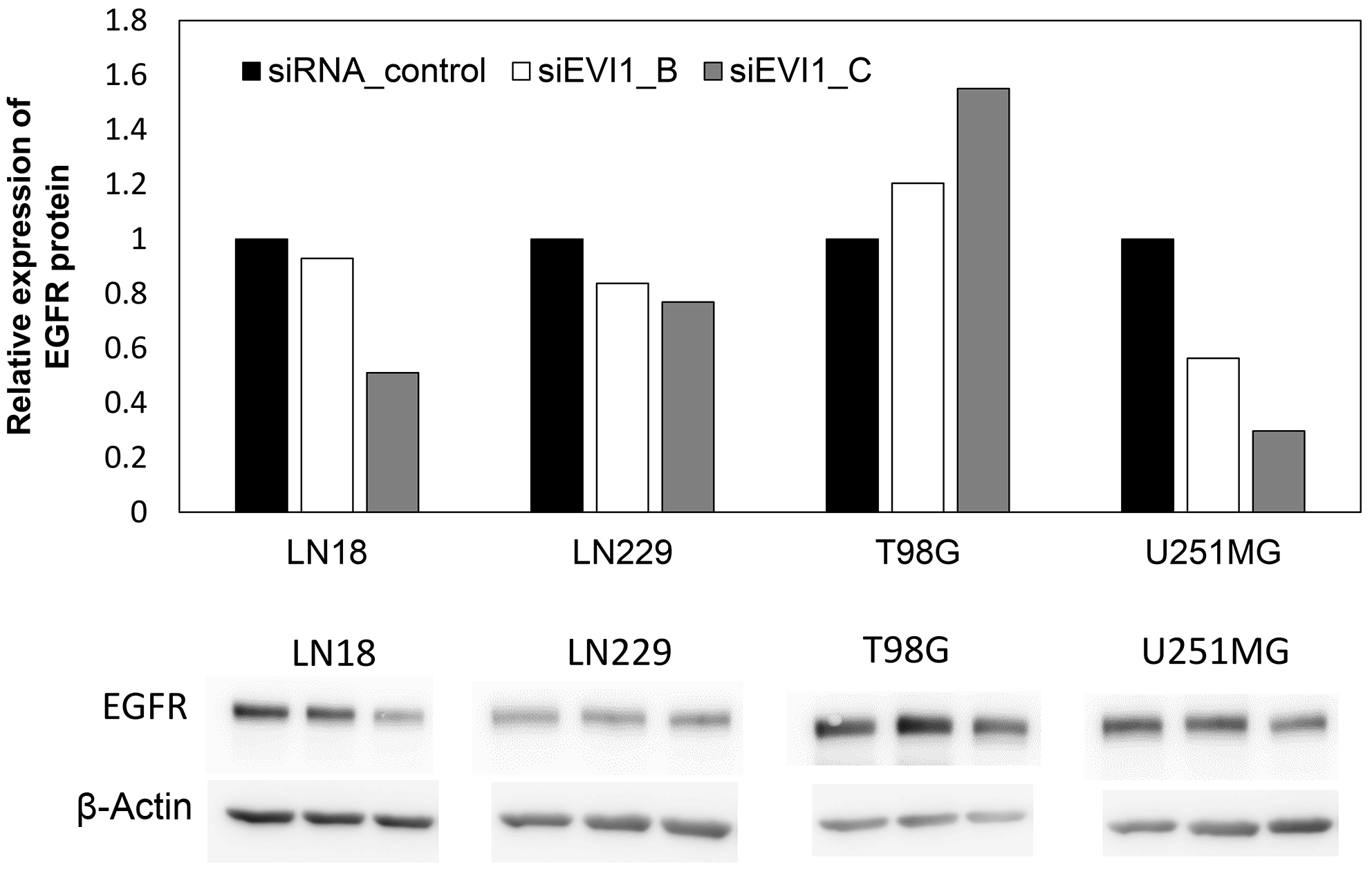

Supplement: Supplementary file 7 — Supplementary file1 (TIFF 271 kb) [file 11060_2019_3310_MOESM7_ESM.tif]

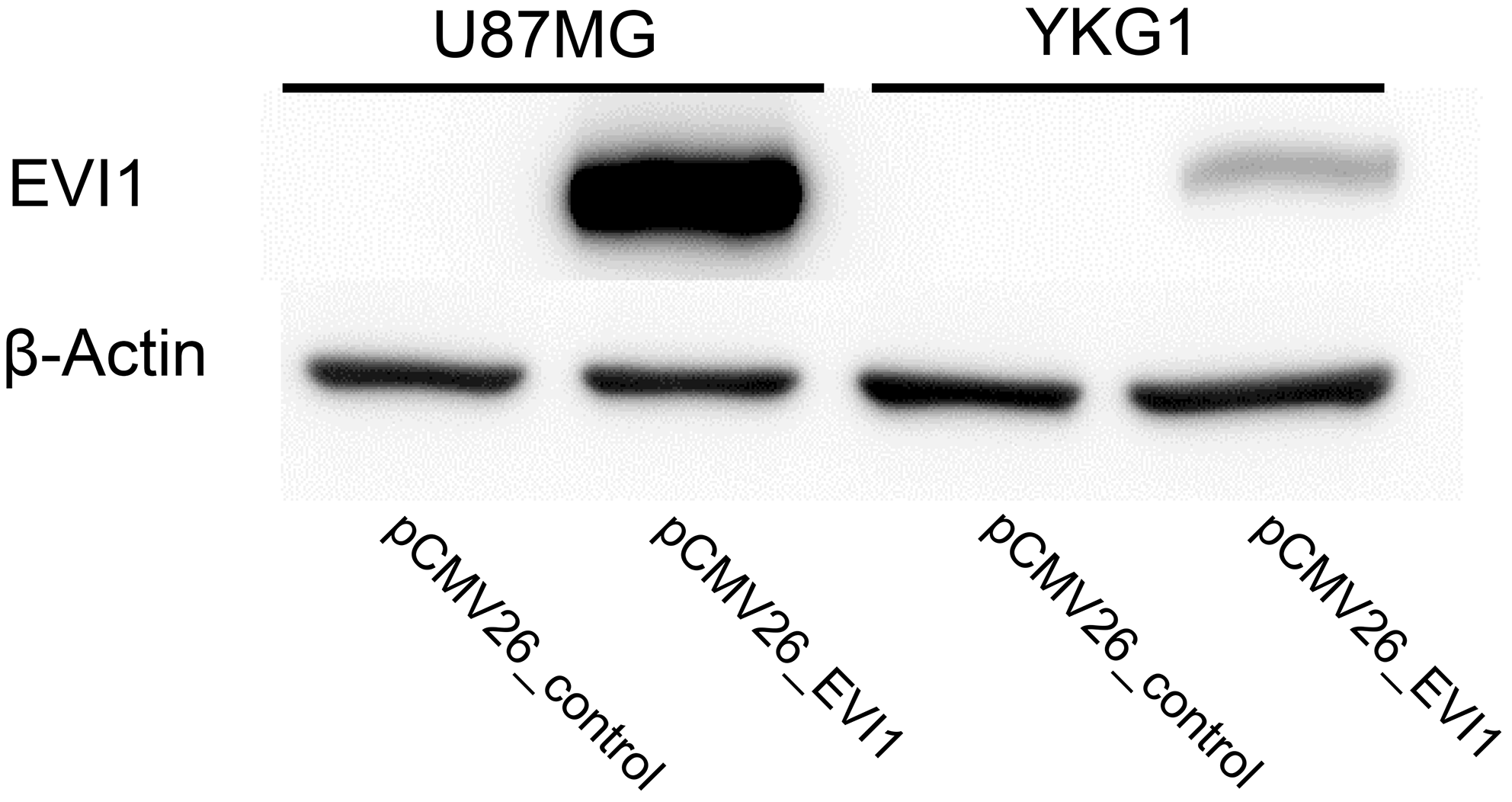

Supplement: Supplementary file 8 — Supplementary file1 (TIFF 645 kb) [file 11060_2019_3310_MOESM8_ESM.tif]
